# EEG-Based Multiword Imagined Speech Classification for Persian Words

**DOI:** 10.1155/2022/8333084

**Published:** 2022-01-19

**Authors:** M. R. Asghari Bejestani, Gh. R. Mohammad Khani, V. R. Nafisi, F. Darakeh

**Affiliations:** Electrical & IT Department, Iranian Research Organization for Science and Technology (IROST), Tehran, Iran

## Abstract

This study focuses on providing a simple, extensible, and multiclass classifier for imagined words using EEG signals. Six Persian words, along with the silence (or idle state), were selected as input classes. The words can be used to control a mouse/robot movement or fill a simple computer form. The data set of this study was 10 recordings of five participants collected in five sessions. Each record had 20 repetitions of all words and the silence. Feature sets consist of normalized, 1 Hz resolution frequency spectrum of 19 EEG channels in 1 to 32 Hz bands. Majority rule on a bank of binary SVM classifiers was used to determine the corresponding class of a feature set. Mean accuracy and confusion matrix of the classifiers were estimated by Monte-Carlo cross-validation. According to recording the time difference of inter- and intraclass samples, three classification modes were defined. In the long-time mode, where all instances of a word in the whole database are involved, average accuracies were about 58% for Word-Silence, 60% for Word-Word, 40% for Word-Word-Silence, and 32% for the seven-class classification (6 Words+Silence). For the short-time mode, when only instances of the same record are used, the accuracies were 96, 75, 79, and 55%, respectively. Finally, in the mixed-time classification, where samples of every class are taken from a different record, the highest performance achieved with average accuracies was about 97, 97, 92, and 62%. These results, even in the worst case of the long-time mode, are meaningfully better than random and are comparable with the best reported results of previously conducted studies in this area.

## 1. Introduction

Brain-Computer Interface (BCI) may be defined as a system that translates brain signals into other kinds of outputs [1]. Electroencephalography (EEG) signals are widely used in the development of the BCI system as well as other investigations regarding information extraction from the brain [2–5].

BCIs are usually concentrated on motor imagery, speech imagination, and image perception tasks. In motor imagery, the imagination of the movement of the hands, feet, eyes, tongue, or other muscles is examined. Usually, there are no physical movements due to disabilities or even absence of the whole body part. EEG signals are used to detect these types of imagination and perform the suitable actions like controlling a wheelchair or moving a robotic arm [6–10].

In speech imagination, also known as Silent-Talk and Silent-Speech, the participants imagine the pronunciation of a particular vowel [2, 11–13], syllable [14–17], or word [18–22] in some defined time intervals. EEG signal during these intervals is processed to determine the imagined word [17, 19, 23–25].

For image perception tasks, the subjects are watching some displayed pictures, for example, simple geometric shapes (rectangle, circle, triangle, etc.), real pictures (persons, animals, planets, objects, etc.), or even written words and letters. The BCI output resembles the type of picture (e.g., the picture is an animal or a planet), kind of the shape (circle or triangle), the person's name or characteristics (known or unknown, male or female, friend or foe, etc.), or whether the shown letter is what the subject has in his/her mind or not. P300 Event Related Potential (ERP) is a well-known and useful feature of EEG signals for these kinds of BCIs [26, 27].

In this study, we examined imagined word recognition for six Persian words and the silence/rest state. The aim was to achieve a suitable classifier structure, which can be used with different number of classes (words), using a minimum processing power for real-time applications. The research process and a summary of some interesting results obtained during the experiment are demonstrated in the succeeding sections.

## 2. Material and Methods

### 2.1. Data Set

#### 2.1.1. Subjects and Instruments

Five male subjects participated in the experiment. They were all healthy, right-handed, and aged between 25 and 45 years. Although the number of subjects is not so high, it is not necessary to incorporate more subjects because all recordings, processing, classifier training, and performance evaluations are done individually for each subject. Interpersonal measures and comparisons are used to assure consistency of the results between different subjects.

**Figure 1:**
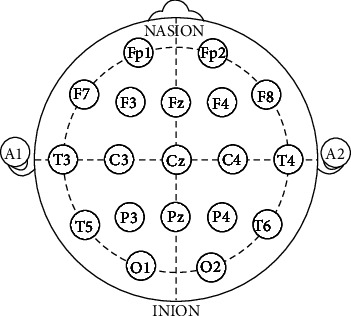
21 electrode locations of the international 10-20 system for EEG recording [28].

To record EEG signals, we used an EEG-3840 EEG set from Negar Andishgan LTD, Tehran, Iran. The system has 21 EEG channels along with 8 external, two ECG, and one EMG channel. Sampling frequency (Fs) was set to 500 samples per second. EEG electrodes were positioned on subject's head and kept in place by a string headset, based on the 10-20 standard (Figure 1).

#### 2.1.2. Test Procedure

**Figure 2:**
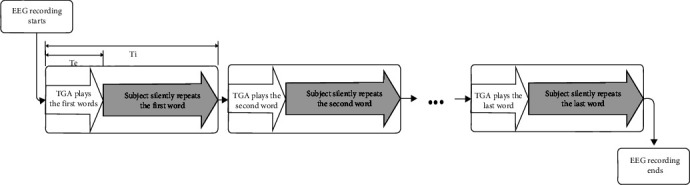
Time sequence of a test's record.

In order to acquire EEG signals during silent talk (imagined word repetition), a test procedure was designed. To implement the tests, we developed a special software, called *Test Generating Application (TGA)*.  Figure 2 illustrates time sequence of tests.

Each “*test*” consists of several (*N*_t_) “*trials*” of some (*N*_c_) chosen “*words*” (or classes) in a pseudorandom order. For example, assume three words *W* = {red, blue, black} with 5 trials (*N*_c_ = 3, *N*_t_ = 5). A single trial has exactly one instance of each word in a random order, e.g., (blue, red, black) or (red, black, blue) and so on. A test (T) is a concatenation of 5 trials (a string of 15 words) like:

*T* = (blue, red, black, red, black, blue, blue, black, red, black, red, blue, red, black, blue).

The TGA program generates these random sets and presents them to the subjects by vocal and/or visual stimulators. In the vocal mode, according to each word instance in the test, a sound or voice is played via earphones. In the visual mode, a shape or word will be displayed on the monitor. The time interval between presenting two consecutive words in a test is called *instance time* (T_i_) and is constant during each test. In visual mode, the time of display of each signal is also a constant parameter (T_d_).

In this experiment, we used vocal stimulation to record EEG signals of imagined words. Subjects were asked to sit on a chair, close their eyes, and listen to the words played on his earphone. When each word played completely, the subject should repeat the word silently for at least 3 times. Closing the eyes helps the subject to concentrate on test procedure. It also eliminates eye blinking, which is the most major EEG artefact. To minimize other kinds of artefacts, the subjects were also asked to be comfort but make no movement by tongue, lips, eyes, or any other organs or muscles. Also, an upper limit was considered for total test time, because elongated test times leads the subjects to become tired and lose their attention.

Subject's EEG signal during every test were continuously recorded and saved to a single file. Also, TGA generates some synchronization signals, which indicates the type and start times of each word instance. These signals were merged into EEG data via external inputs of EEG-3840.

#### 2.1.3. Data Set Structure

We selected six Persian words for this experiment: {خیر، بله، راست، چپ، پایین، بالا}. They are pronounced as {(bʌlʌ), (pʌyɪn), (chæp), (rʌst), (bæle), (kheɪr)} and are equivalents of {Up, Down, Left, Right, Yes, No}, respectively. These words have been selected for several reasons, such as the following:

- They are complete and meaningful Persian words
- They can be used for navigating a mouse/wheelchair or filling a simple questionnaire form
- Half of them (chæp, rʌst, and kheɪr) have only one syllable, while the others have two
- We can divide them into three pairs with opposite meanings, i.e., {Up, Down}, {Left, Right}, and {Yes, No}
- The pairs also have all three possible combinations of syllable counts, (two, two), (one, one), and (one, two)
- The same pairing combines the two words, which are usually used together

Another special item, the Silence, was also added to the above word list to indicate the “rest” or “no word processing” state of the brain.

Playing (or repeating) time for each of selected words with normal speed is about 500 to 1000 mSec, so we considered instance time to be 4 seconds (T_i_ = 4000). If we limit the total test time to be about 4 minutes, then each test can contain 60 word instances. Thus, the six words were divided into three tests, each with two words and the Silence (*N*_*c*_ = 3) and set *N*_*t*_ = 20 trials. Specifically, there were three tests:

(T1) *W* = {Up, Down, Silence}; *N_t_* = 20; *T_i_* = 4000

(T2) *W* = {Left, Right, Silence}; *N_t_* = 20; *Ti* = 4000

(T3) *W* = {Yes, No, Silence}; *N_t_* = 20; *T_i_* = 4000

Each subject incorporated in five “sessions,” each in a single day with at least a one-week interval. Each session consisted of two “parts” separated by a short break. In any part, all three tests were recorded so that all of the selected words were used in every part.

Finally, the raw EEG data set consists of 30 records (5 sessions × 2 parts × 3 records) for every subject, containing 200 instances (5 sessions ∗ 2 parts ∗ 20 trials) for each of the six selected words and 600 instances (200 ∗ 3 tests) of “Silence.” Each record had19 EEG channels, with two additional sync channels sampled at 500 Hz with a 16-bit accuracy.

### 2.2. Preprocessing

**Figure 3:**
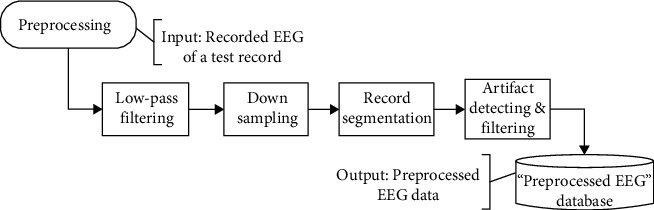
Preprocessing steps.

All the recorded data were preprocessed in offline and EEG data for each word instance was saved as a separate record in *Pre-processed EEG Database*. Preprocessing consists of following steps (see Figure 3).

#### 2.2.1. Low-Pass Filtering

Frequency bands of EEG signals spans from DC to Gamma band (above 30 Hz). Sampling frequency (*F_s_*) was set to 500 Hz, thus the recorded EEG signals had frequency components up to 250 Hz, which is far beyond the useful EEG frequency bands. In order to suppress higher frequencies and specially the power line noise (50 Hz), we used a 0-32 Hz low-pass filter (LPF).

#### 2.2.2. Subsampling

After removing high frequency components, we down sampled the EEG signals from 500 Hz to 100 Hz by replacing each five consecutive samples with their average. This reduced the size of the recorded data to 1/5 of its original size and improved the speed of subsequent processes.

#### 2.2.3. Record Segmentation

As mentioned above, each continuous record of EEG signals, consisted of *N*_*i*_ = 20 instances of two words and the Silence. In this step, EEG data of word instances were separated from each other. This was done by using the “Sync Data”, which was generated by TGA and saved in separate channels of EEG signal.

Although all word instances were assumed to have the same time length (*T*_*i*_ = 4 sec), EEG signals for each segment were cropped (or padded with zeros) to have exactly the same number of samples.

#### 2.2.4. Artifact Detection and Filtering

EEG signals may always be corrupted by various kinds of artefacts such as blinking, eye movement, wanted or unwanted movements, and so on. In our experiment, due to closed eyes during EEG recording, the major artefacts of blinking and eye movement have been minimized. Moreover, subjects had been asked to control and avoid any additional movement or muscle activity during recordings.

Besides all of these, average energy of EEG signals during an instance has been considered as a measure of signal quality. Any instance with an energy much above or below the overall average energy of whole recorded signal, has been filtered out. Total number of rejected instances was less than 2% of all recorded EEG data.

### 2.3. Feature Extraction

**Figure 4:**
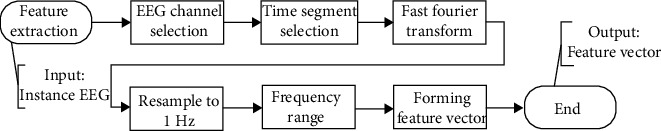
Feature extraction.

The feature vector used in this research was simply the amplitude of each single frequency in selected EEG channels. The advantage of frequency-related parameters is that they are less susceptible to signal quality variations, which may be present due to electrode placement or the physical characteristics of subjects [5].  Figure 4 shows the feature extraction steps.

#### 2.3.1. EEG Channel Selection

Depending on the application and features needed, only a selected set of EEG channels are used for processing, which may be one, all, or any other combination of the channels. For the results presented in this article, we have used 19 EEG channels from 21 available EEG channels in 10-20 standard (see Figure 1). Channels A1 and A2 were not used because they are not actually scalp EEG channels. Instead, they are often used for contralateral referencing of all other EEG electrodes [28].

#### 2.3.2. Data Time Segment Selection

**Figure 5:**
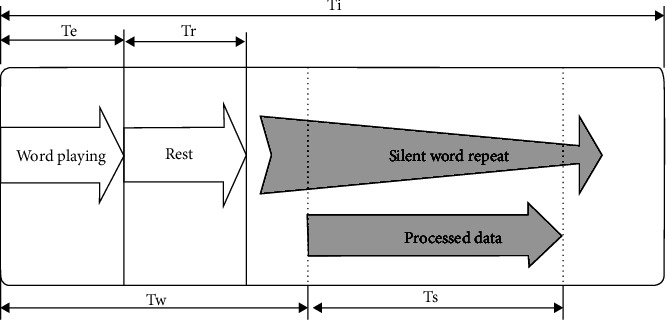
Process time selection.

The total time duration for signals of each instance of a word was *T_i_* (4 seconds). The first portion of this time (*T_e_*, up to 500 mSec) corresponds to excitation (hearing the played word). Then, after a small rest time (*T_r_*), the subject repeats the word silently for a few times. As illustrated in Figure 5, we only processed the EEG samples for an interval *T_s_*, starting after *T_w_* from beginning of the instance. Obviously, *T*_*w*_ ≥ *T*_*e*_ + *T*_*r*_ and *T*_*s*_ ≤ (*T*_*i*_ − *T*_*w*_). Experimentally, we selected *T*_*w*_ = 1 and *T*_*s*_ = 2.5 seconds.

#### 2.3.3. Fast Fourier Transform (FFT)

To compute frequency components, we used absolute value of FFT of EEG signals. Furthermore, the amplitudes of these absolute FFTs were normalized by dividing them to their maximum value.

#### 2.3.4. Resample to 1 Hz Resolution

Time duration of the signals is *T_s_*, therefore, the initial resolution of their spectrum is *1/T*_s_. To normalize the length of feature vectors against sampling frequency and duration of the signals, we resampled FFTs to one Hertz resolution. This typically reduces the length of feature vector. Besides, since the signals were already filtered with a 32 Hz low-pass filter, only first 32 values of this resampled FFTs had valid amplitudes.

#### 2.3.5. Frequency Range Selection

Usually, not all of the signal frequency range are used. For example, one may filter out EEG Delta and Gamma bands by omitting first four and last two values of resampled FFT, corresponding to frequencies below 4 Hz (0 to 3) and above 30 Hz (30, 31). In this experiment, all frequency components were used, except the first (DC) value.

#### 2.3.6. Forming Feature Vector

Finally, the selected FFT values (of all selected channels) were concatenated to form instance feature vector. If *N_ch_* channels and *N_f_* frequency values were used, feature vector length would be *L*_*f*_ = *N*_*ch*_∗*N*_*f*_.

### 2.4. Classifier Structure

We used binary (2-class) support vector machines (SVM) as the basis of our classification method. One SVM was trained to classify between 2 specific classes consisting of a pair of selected words or a word and the Silence. Let SVM (*i, j*) be the machine trained for two classes *i* and *j*. Obviously, if an arbitrary feature set is presented to such a machine, it would be classified to one of the trained classes (*i* or *j*), even though it may belong to neither.

**Figure 6:**
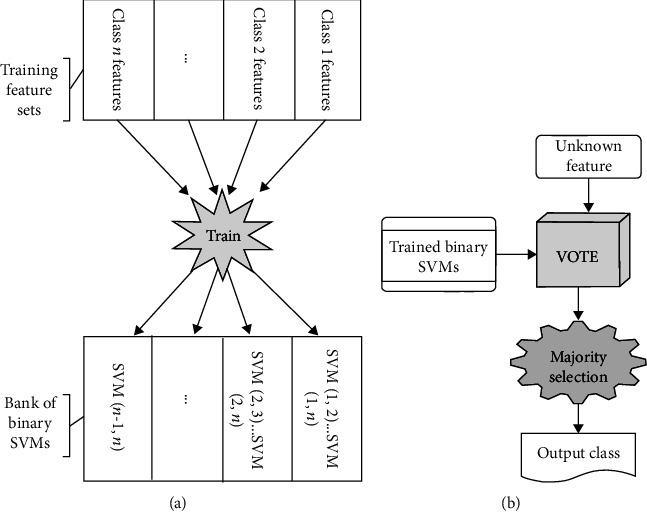
(a) Training of binary SVM machines and (b) classification algorithm.

For n-class classification, there would be *n*(*n* − 1)/2 of such classifiers. *Majority Rule* was used for winner selection. Besides training, classification consists of the following steps (Figure 6):

- The unknown feature set is presented to all binary classifiers
- If a relative majority of the classifiers vote for a single class *k*, then class *k* wins and the features are assigned to this class
- If two classes, namely *k* and *l*, had the same maximum vote counts, then the class voted by SVM (*k,l*) wins
- If more than two classes had the same maximum vote counts, then output class is undefined and the input feature would be tagged to belong to special class “Unknown”

Note that if we assume that every binary classifier has a relatively good performance, then this Majority Rule is also *the Rule of Specialists*. For example, in the special classification case of two words and the silence (W1, W2 and S), if the input sample is from the S class, then the two machines SVM (W1, S) and SVM (W2, S) will realize it and the sample will be correctly assigned to S, regardless of the vote of SVM (W1, W2) machine. On the other hand, if the input belongs to one of the words' classes, then the former two machines reject the S and vote for W1 and W2, respectively. Now the third machine, the words specialist SVM (W1, W2), would determine the correct class.

### 2.5. Evaluation Procedure

#### 2.5.1. Performance Measurement

To evaluate our classifier, Monte-Carlo cross-validation was used. EEG data of proper classes were fetched from “Pre-processed EEG Database” and their features were extracted.

In each round (*r*) of the Monte-Carlo simulation, instant feature sets were randomly divided into two partitions: the train set, with approximately 70% of samples and the test set, with the rest 30%. As usual, SVMs were trained with train set data and used to classify features in the test set. A confusion matrix (*C_r_*) was formed with known (true) classes as rows and resulted classes from the classifier as columns, so that the number in (*i*, *j*)-th element of the matrix is the number of features from class *i*, classified as class *j*. As mentioned above, in the case of multiclass classification, an extra column, “Unknown”, should be added to *C_r_*. In every iteration, the accuracy of classification was computed:

(1) $$\begin{array}{c} Ar=\frac{\text{Number of correct classifications}}{\text{Total number of classifications}}=\frac{\text{trace }\left(Cr\right)}{\text{sum }\left(Cr\right)}, \end{array}$$

where trace (*C_r_*) and sum (*C_r_*) are sums of main diagonal and all elements of *C_r_*, respectively. When all the Monte-Carlo iterations were done, the final confusion matrix can be computed by the sum of all iteration matrices:

(2) $$\begin{array}{c} \text{C}=\overset{Nr}{\underset{r=1}{\sum }}Cr. \end{array}$$

In (2), *N_r_* is number of Monte-Carlo iterations. The final classification accuracy and its standard deviation were estimated by the following:

(3) $$\begin{array}{c} A=\frac{1}{Nr}\overset{Nr}{\underset{r=1}{\sum }}Ar\text{ and }\sigma =\sqrt{\frac{1}{Nr}\overset{Nr}{\underset{r=1}{\sum }}{\left(A-Ar\right)}^{2}}\; \end{array}$$

Alternatively, *A* can be calculated by eq. (1), with *C_r_* replaced by *C*. Values of *A_r_* were kept in a column vector called Class Measures (*M_c_*) for further evaluation extensions.

#### 2.5.2. Data Representation

For simplification, all names (the words or class names, EEG channel names, records, etc.) were coded with integer numbers. These codes have been used in all figures and tables of this report.

Recording parts were numbered in chronological order so that the first part of first session (the oldest part) is called part-0 and the second part of the last session (the newest) is part-9. Similarly, every word was presented in 10 records, one record in every part, except the silence, which was present in all records. So, we may refer to part numbers of a word as “record number.” We also considered all instances of silence in a part as a single record.

Table 1 shows the class names and their code numbers. As seen in this table, each word has up to 11 class numbers. If all instances of a word in the whole database is considered, the code will be a single digit number (from 1 to 7 for six words and the silence). When only samples from a specific record have to be referenced, the code should be followed by the part number. For example, instances of word “kheɪr “(class 4) in the second record (part no 1) have class no 41.

### 2.6. Classification Modes

EEG signals are not stationary, therefore the time differences between the recording times of EEG signals can directly affect the performance of classification. Based on recording time difference of inter- and intraclass samples, three *modes* of classification were distinguished in this research:

- *Long-time classification* where all instances of a class in whole database are involved
- *Short-time classification* where only instances of the same record (or part) are used
- *Mixed-time classification* where samples of every class are taken from a different record

In the first mode, the difference in the recording time of samples of the same class spans from a few seconds to several weeks. This is the time during which the EEG signals of a subject were recorded. The same is true for interclass time differences.

In the second mode, intraclass time difference is at most as short as a test time (4 minutes). The interclass difference would be at most equal to duration of a recording part which was kept in about half an hour.

In the third mode, the intraclass times is the same as the second mode, while the interclass time is typically many days (about an hour at least and some months at most).

## 3. Results Evaluation

**Figure 7:**
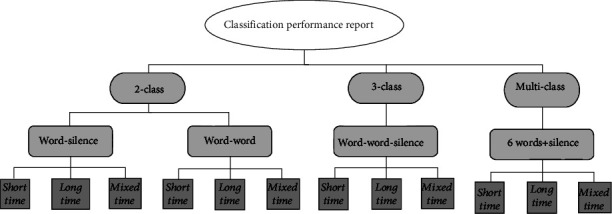
Organization of evaluation results.

We tried several classification cases with our captured data sets, selected feature extraction methods, and designed classifiers. Some interesting results are presented below. First, the results of the simplest case of a 2-class classification are reported, then 3-class case, and, finally, some examples of the results in multiclass classifications are mentioned. In all cases, the above three classification modes are considered (Figure 7).

To avoid making the manuscript lengthy, feature extraction parameters are kept constant during this report. The selected set of parameters are shown in Table 2. It is almost optimal [29], but there may be other combinations, which produce comparable results. Furthermore, otherwise specified, the results are average of all subjects.

### 3.1. Two-Classes Type

First, we examined the simple case of a two-word classification. In this case, there was only one SVM which was trained with features extracted from two specific classes. Different combinations of classes and/or feature parameters were examined.

#### 3.1.1. Word-Silence

This type of classification is important for discriminating talking/not-talking states, especially in real-time silent-talk applications.  Table 3 summarizes some results of classifying EEG signals corresponding to silent repeating of a single word, chosen from the above six-word set, and the “Silence” case.

*(1) Short-Time Classification*. To explain the accuracies in Table 3, we start with classifying between two classes 32 and 72 (instances of the word “Left” and the Silence in record no. 2, or L2 and S2, see Table 1). Here, we only work with EEG data of subject no. 1. When validating this SVM classifier by the Monte-Carlo cross-validation method, confusion matrix (*C_r_*) is calculated in each iteration, and accuracy (*A_r_*) is estimated by eq. (1). At last, the final confusion matrix (*C*) is obtained by eq. (2) and all (*A_r_*) are concatenated to form a column vector named *M_c_* (see Section 2.5.1).

**Figure 8:**
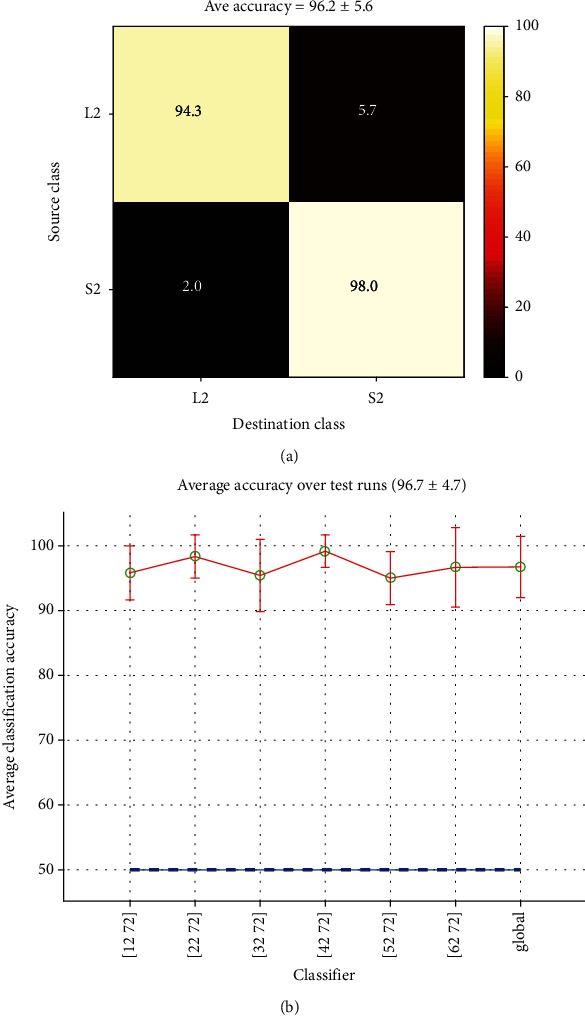
(a) Confusion matrix for [32 72] classification and (b) estimated classification accuracies for each Word-Silence pair in record No 2. The last value is the global performance.

Figure 8(a) shows a typical confusion matrix (*C*) for this classifier. The values in this matrix were calculated from the results of 30 Monte-Carlo iterations. Element colors are graphical illustrations of their values. As mentioned, the value in row *i* and column *j* is the percent of samples from class *i*, classified as class *j*. For example, 94.3% of total test samples of the word “Left” were correctly identified, but 5.7% of them were falsely classified as “Silence.” From eq. (3) and data in *M_c_*, the average accuracy for this classifier was *A* = 96.2% with a standard deviation *σ* = 5.6%.

We repeat the same procedure for all the six words in record no. 2 (classes 12, 22, 32, 42, 52, and 62 against class 72) and concatenate all (*M_c_*) into a matrix *M*. The average accuracy for each word classifier and its standard deviation can be estimated from columns of *M*. We can also calculate the global mean and standard deviation for all data in *M* as a measure of performance for the task of discriminating a word from silence in this record. These values are shown in error-bar of Figure 8(b). In this figure, green circles show classifier's average accuracy and red bars indicate the standard deviation around the mean. The last bar is the global mean and standard deviation. The horizontal dashed blue line shows the chance level accuracy, which is 50% for balanced binary classification. Regarding the definition of classification modes (Section 2.6), this is a short-time classification because only instances of words and silence in the same record are used.

A better estimation for the accuracy can be obtained by averaging the performances over all records and all subjects. This is the value recorded as the accuracy of short-time Word-Silence classification, in the first row of Table 3 (95.7 ± 8.3%).

**Figure 9:**
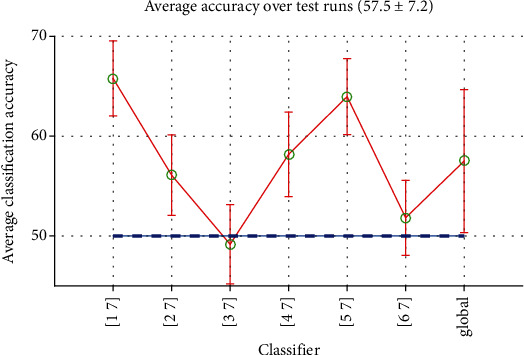
Performances of Word-Silence classifiers in long-time mode with their global average.

*(2) Long-Time Classification*. In the long-time mode, all instances of a word and silent from each particular subject are used to train and test the classifier.  Figure 9 shows the performances of classifiers in this case. As seen, the classifiers have generally a poor performance and in some cases they even did not better than chance. The global accuracy is weakly above 50%, thanks to the relatively good performance of (1, 7, 5, and 7) classifiers.

**Figure 10:**
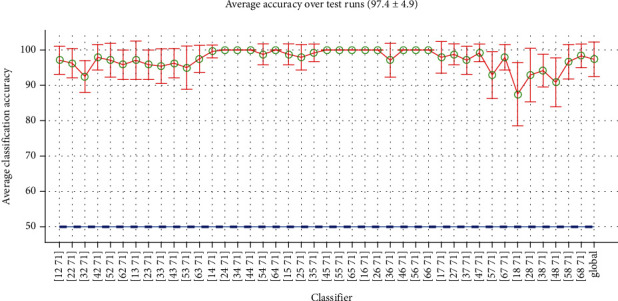
Performances of some Word-Silence classifiers in mixed-time mode and their average.

*(3) Mixed-Time Classification*. In mixed-time mode, every class data should be picked from a separate record of the same subject, e.g., the word “Up” of the third record (class no. 12) and “Silence” from the second record (class no. 71). Hundreds of such classifiers can be defined for every subject.  Figure 10 shows performances for some of these Word-Silence classifiers in mixed-time mode using EEG data of subject no. 1. As may be expected, classification performance in this mode is generally better than both previous modes. In some cases, accuracy reaches to 100%, and, in a worst case, it remains above 80%. The global average accuracy is also slightly better than the short-time mode.

#### 3.1.2. Word-Word

For this case, there was also one SVM machine, trained with feature vectors of instances of a selected pair of words. The procedure and results in this case were the same as Word-Silence, with the silence replaced by a word. Three classification modes were considered, but since there were several words which can be picked up for the second class, the total number of possible combinations was much more than the previous case.

**Figure 11:**
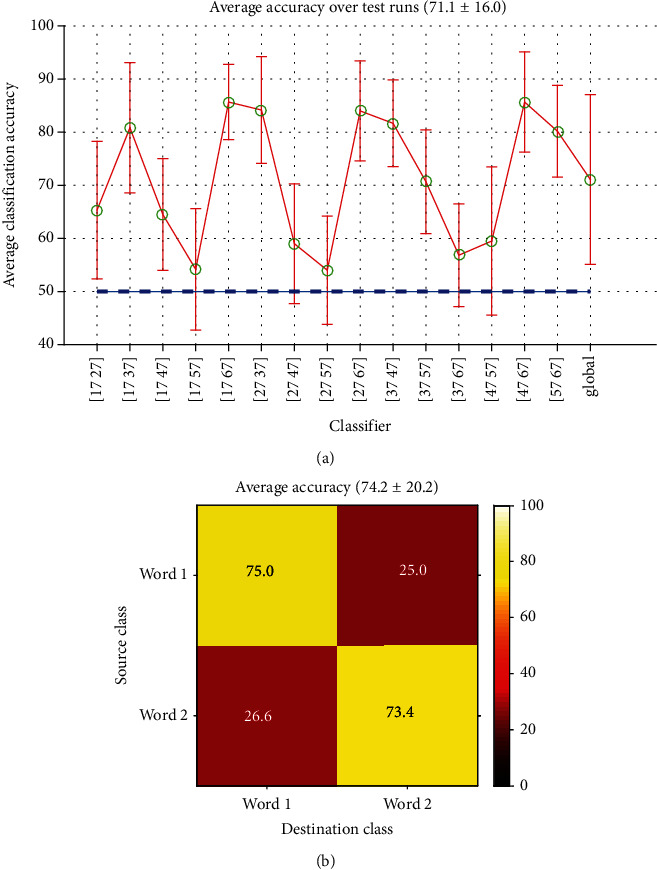
Short-time Word-Word classification: (a) accuracies estimated for record no. 7 and (b) confusion matrix (average).

*(1) Short-Time Classification*. In this case, words instances of a recording part were compared together. In each part, there were15 (6∗5/2) possible combinations.  Figure 11(a) shows the accuracies estimated for record no. 7of subject no. 1. There were seven classifiers with relatively good performance (80-90%), three were moderate (60-80%), and the other five had poor accuracies (50-60%). Three of the weakest results belonged to classifiers (17, 57), (27, 47) and (37 67), specifically classifiers with words (Up, Down), (Yes, No) and (Left, Right). Note that these are the words which were paired into three designed tests T1, T2,and T3 (sec.  2.1.3) and therefore had the smallest interclass time difference, nearly the same as their intraclass distance (a few seconds up to less than 4 minutes).

The confusion matrix for this type of classification is shown in Figure 11(b). Global average accuracy was about 74.2 percent with a relatively large standard deviation (*σ* = 20.1), which means a possible but not so reliable classification.

**Figure 12:**
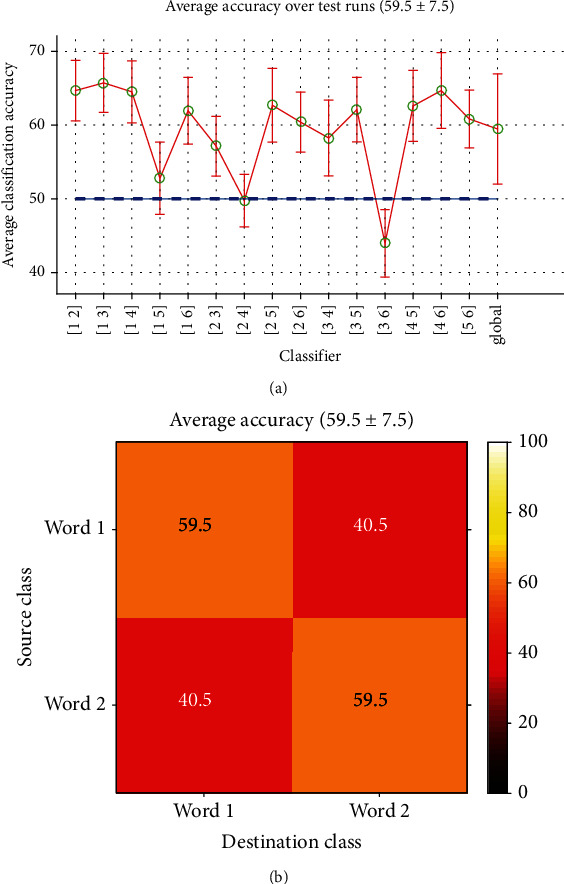
Long-time Word-Word classification: (a) accuracies of all classifiers and (b) confusion matrix (all classifiers average).

*(2) Long-Time Classification*. Using the long-time data reduces the total iterations needed to estimate Word-Word classification performance for each subject to 15. The results are illustrated in Figure 12(a). Although the accuracy levels have decreased compared to Figure 11(a), the graph shapes are roughly the same: three classifiers with chance level accuracies, a few in middle, and a majority in higher levels of accuracy. The smallest accuracies belonged to the same classifiers working on words which were paired in tests.

Figure 12(b) is the average confusion matrix for all classifiers. The average accuracy is about 60% with a standard deviation equal to 7.5 which again has a breaking higher performance than random classification.

**Figure 13:**
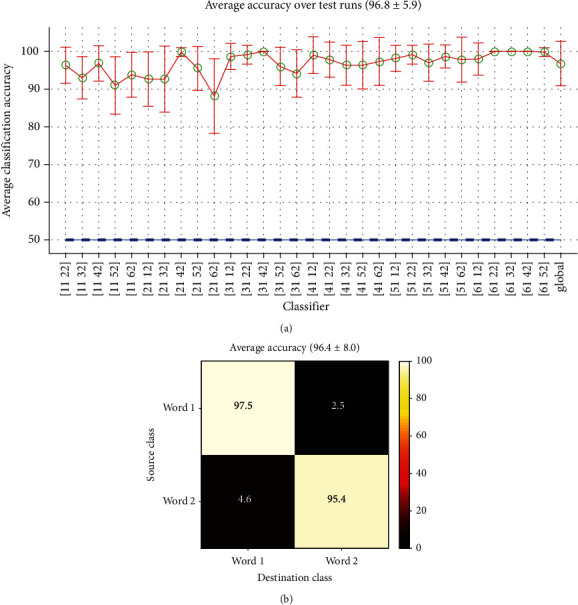
Mixed-time Word-Word classification: (a) accuracies estimated for record no. 1 vs record no. 2 and (b) average confusion matrix (all classifiers).

*(3) Mixed-Time Classification*. In mixed time, each word in a record are compared with other words in another record. Classification results of words in record no. 1 against record no. 2 of subject no. 1 have been shown in Figure 13(a). Nearly all classifiers have excellent performance compared with other classification modes (90-100%). The overall accuracy, calculated by averaging over thousands word/record combination was 96.4% with *σ* = 8.0. Average confusion matrix is shown in Figure 13(b).

Table 4 summarizes overall accuracies for Word-Word classification in all the three modes. Compared with the corresponding values for Word-Silence classification, accuracies were only slightly reduced in long- and mixed-time modes. In the short-time mode, performance reduced noticeably due to low accuracies in discriminating words which were paired in tests.

### 3.2. Three Classes

In the three-class classification, there are three possible classes (*C1*, *C2*, and *C3*) and the classifier consists of three binary SVMs, (*C1*, *C2*), (*C1*, *C3*), and (*C2*, *C3*). If a sample with unknown class *C* is presented to the three SVMs, then with the majority rule which we have selected for the classifier (see Section 2.4), there would be only two possible states:

- If two classifiers agree on their common class (*Cx*) then the unknown sample will be assigned to this class (*C* = *Cx*). Of course the third machine cannot agree with this choice
- If every SVM has its own choice, not common to any other machine, then the sample *C* will remain unknown and will be assigned to the class of “Unknowns” (*C0*)

An important case of this classifier type is the two words and silence classifier (WWS).  Table 5 shows the summarized results for three-class WWS classification in all three modes.

#### 3.2.1. Short-Time Classification

**Figure 14:**
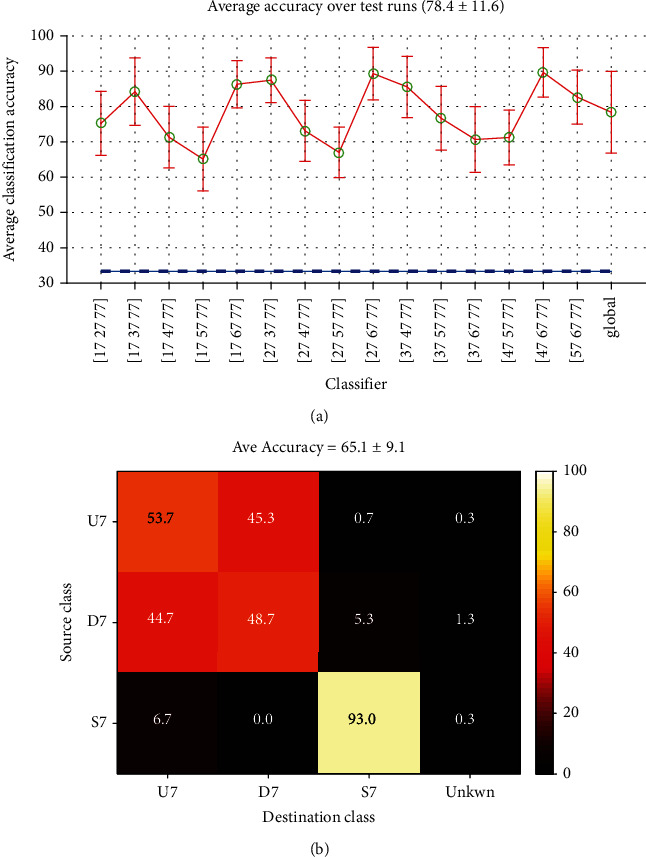
Short-time Word-Word-Silence classification, (a) accuracies estimated for record no. 7, (b) Confusion matrix of the worst case in record 7 (17, 57, 77).

Figure 14(a) shows the classification results of WWS classification on EEG data from record no. 7 of subject no. 1. The Word-Word classification results of this record were shown in Figure 11(a). The shape of the two curves are very close together, but the accuracies in WWS case are slightly greater than the two-class case with the same words. Furthermore, the chance level in three class case is about 33% compared with 50% in binary classifiers. These made the resulting performance to be much better than Word-Word classifier.

Besides the overall good quality of classification, the worst cases were again the classifiers which worked on the words with the minimum interclass time difference.  Figure 14(b) shows confusion matrix of the worst classifier in record no. 7, the (17, 57, 77) or more clearly the (U7, D7, S7) classifier. It is clear that the classifier almost dose not distinguish between the two words but has a good performance for the silence.

**Figure 15:**
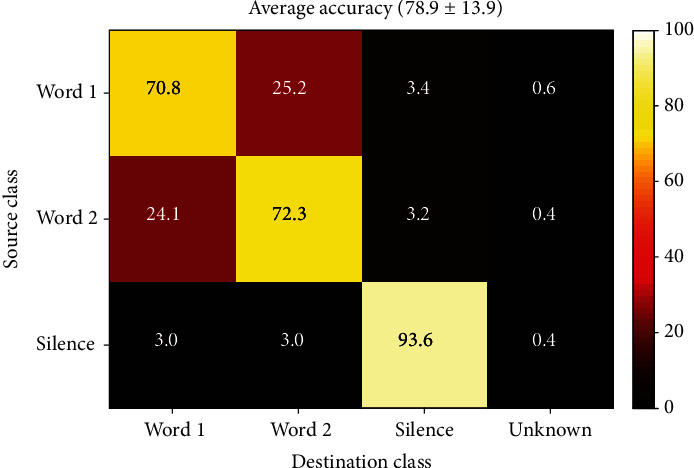
Short-time Word-Word-Silence classification confusion matrix (overall average).

The global average accuracy was calculated for all short-time mode WWS classifiers of all records whose results indicated a rate of 78.9 ± 13.9% which is shown with its confusion matrix in Figure 15.

#### 3.2.2. Long-Time Classification

**Figure 16:**
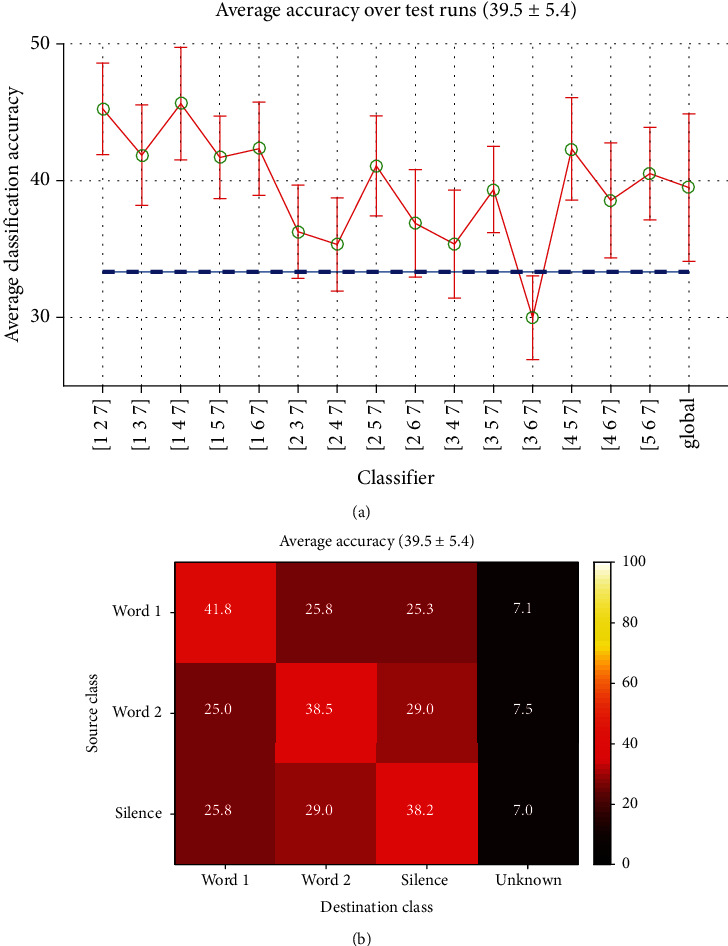
Long-time estimated Word-Word-Silence classification, (a) accuracies, (b) confusion matrix.

Here the classifiers are defined over three different words in the whole recordings.  Figure 16(a) shows the accuracies of all 15 classifiers in this category. The accuracies were generally low (all below 50%) and in some cases were close to random decision. The overall accuracy was above the chance by a very narrow margin (39.5% with *σ* = 5.4).  Figure 16(b) is the average confusion matrix of all classifiers.

#### 3.2.3. Mixed-Time Classification

**Figure 17:**
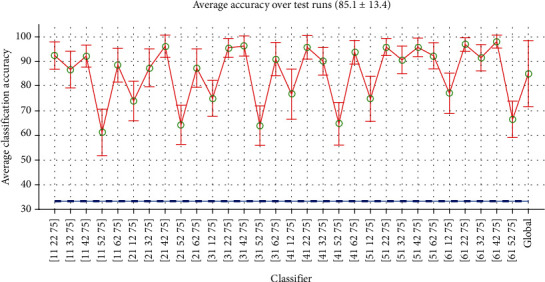
Mixed-time Word-Word-Silence classification accuracies estimated with words from records no. 1 and 2 and silence form record no. 5.

Figure 17 shows some results for mixed mode three class WWS classification. The words combinations was the same as Figure 13(a) where the first word was picked from record no. 1 and the second word comes from record no. 2 of subject no. 1. Silence samples were from record no. 5.

As seen in most cases, the classification accuracy was well above 85%. When second word was taken from class no. 12 the accuracy reduced to about 75% and if the word was from class no. 52 the accuracy again decreased further to 60-70%. This shows a low performance for (12, 75) and (52 75) SVM machines. The average accuracy in about 85% which is much lower than 98% of Word-Word case.

**Figure 18:**
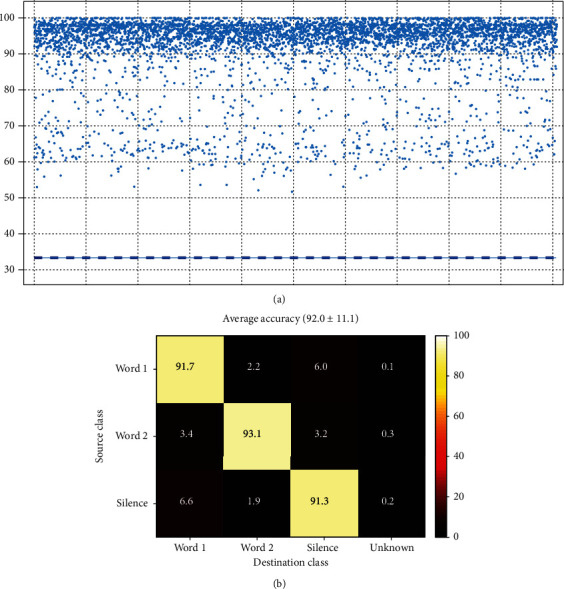
Mixed-time Word-Word-Silence classification: (a) accuracies of thousands of WWS classifiers with unique combination of classes and (b) average confusion matrix for all classifiers.

Figure 18(a) shows a scatter of accuracies of several thousands of WWS classifiers with a unique combination of classes.  Figure 18(b) is the overall confusion matrix of classifiers. The global average accuracy of all classifiers was about 92% which is not as good as Word-Silence and Word-Word classifiers but is still a good result. Note that in about 0.6 percent of cases a feature set could not be classified.

### 3.3. Multiclass Classification

#### 3.3.1. Short-Time Classification

**Figure 19:**
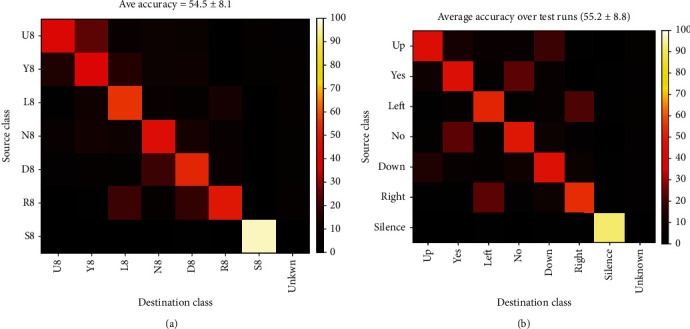
Confusion matrix of multiclass classifier of all words in a record: (a) record no. 8 and (b) average of all records.

As the first example for multiclass classification, here we take a quick glance at the classification of all the seven classes in a single record. Obviously, this is a short-time classification mode with seven classes. The classifier has 21 SVM machines which will select the output class by the Majority Rule.  Figure 19(a) presents the confusion matrix of such classifier for all words in record no 8 of subject no. 1. The average accuracy was about 55%. The silence had the maximum single class accuracy (about 95%), but no other single word accuracy exceeded 50%. The largest error rates were between the words paired into a test, as seen, for example, in discrimination of L8 and R8. Note that the random selection accuracy in this classifier was1/7 or about 14 percent.  Figure 19(b) is the average confusion matrix for all records. The average accuracy was again about 55% and ranged from 47.5% to 65.1% for single records.

#### 3.3.2. Long-Time Classification

**Figure 20:**
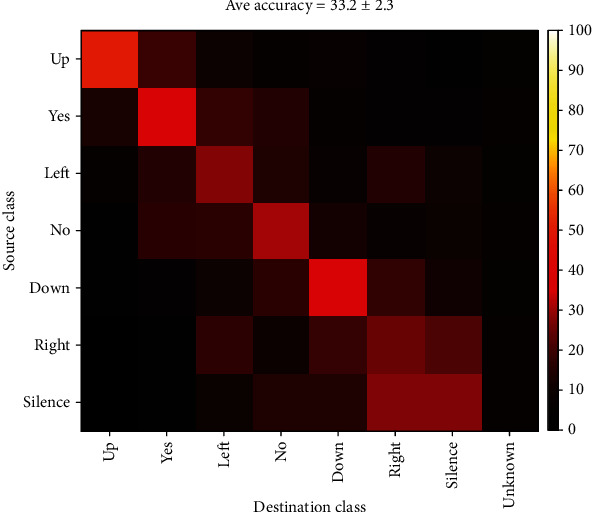
Confusion matrix for long-time classification of all 7 words.

This case is similar to the short-time case except that similar words from all records are tagged as a single class. The accuracy of classification in this case was about 32%. Although this accuracy is about 2 times the chance level, it cannot be considered a good result. Once again, the highest error rate belonged to “Left” and “Right” classes.  Figure 20 shows the confusion matrix of this classifier.

#### 3.3.3. Mixed-Time Classification

**Figure 21:**
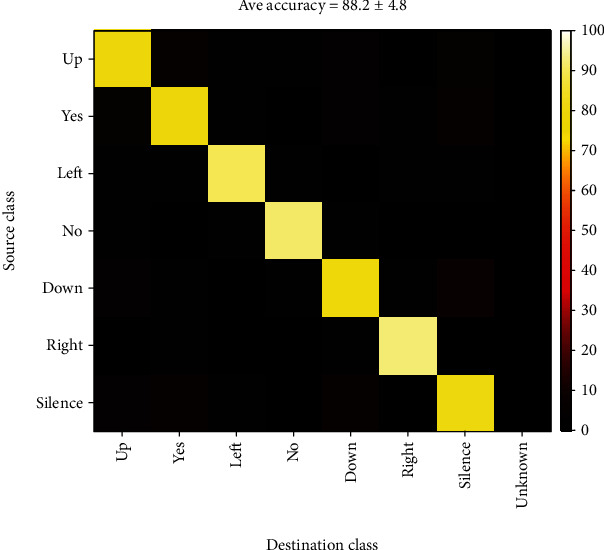
Confusion matrix for mixed-time classification of all 7 classes.

For the classification of all the seven classes in mixed-time mode, we should choose every class sample from a different record. Since there are hundreds of thousands of ways (10!/3! = 604800 per subject) to do this, the full estimation of classification accuracy by averaging all possible cases is impractical. Therefore, to calculate the confusion matrix shown in Figure 21, we used the average of 100 randomly selected permutations for each subject.

The overall accuracy was more than 88 percent, which is much better than both short-time and long-time modes.

**Figure 22:**
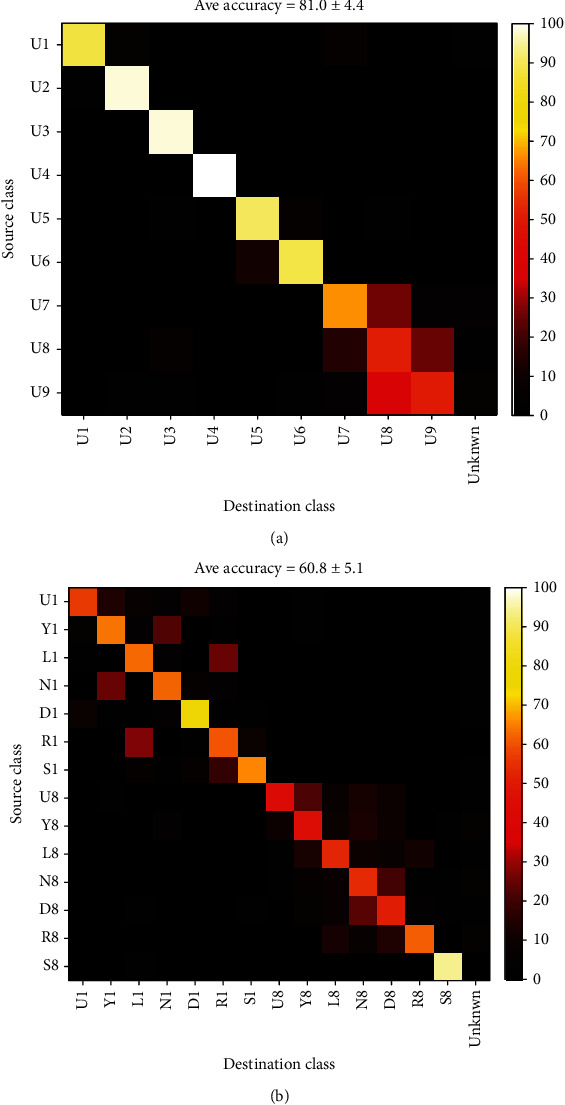
Confusion matrix of mixed-time multiclass classifications: (a) a single word in different records and (b) all words in 2 records.

Another interesting case of mixed-time multiclass classification was classifying the instances of a word in a record against the same word in other records.  Figure 22(a) shows the confusion matrix of a classifier for the word “Up” in records no. 1 to 9 of subject no. 1. Note that how good the words in different records are distinguished.  Figure 22(b) is a 14-class classifier of all 7 words in two different records. We can see that the two records have almost completely separated so that second and third quarters of the graph is almost empty. Consequently, in each part, words of a record are classified with a confusion pattern very similar to classifying the same record alone (compare patterns in Figure 19(a) with lower right section of Figure 22(b)).

## 4. Discussion

At first, it is worth noting that all presented algorithms and parameters, are the results selected after the evaluation of many (hundreds in some cases) possible substitutions. For example, many combinations of EEG-Channel collection and montages, tens of feature extraction methods and parameters, and a few classifier types and parameters are evaluated. Some had poor absolute results like Artificial Neural Networks (ANN) as classifier or raw EEG signal amplitudes as features. Some had weaker results than those reported here, e.g., Principle Component Analysis (PCA) features with a Minimum-Distance classifier [30], or using Banana montage for EEG signals [31]. Some did not fulfil our requirements, for example, Random Forest Classifier (RFC) needs to be separately configured and trained for each class combination. Also some combinations or parameters did not notably affect the performance and were left in their default values or set to an arbitrary value, like SVM machines basis function which was “Linear” by default (in MATLAB® version we used) or ordering of channels in EEG signal.

Table 6 shows a summary of average accuracies in all classification types and modes described in this report. As seen in each column of this table, the minimum accuracy for each type of classification accrues in long-time mode, where the data of a relatively long-time interval were merged together. This is mainly due to the fact that EEG signals are not stationary and their statistics (or properties) change along the time. In long-time mode, samples from different times and with different properties are got together in a single class. The complexity and diversity of such large classes, causes the SVM machines to not precisely distinguish between them. This is evident in increased learning time of the machine as well as its learning accuracy. Hence, it can be concluded that we should not use (too) old EEG data in a silent-talk BCI application.

In the 2-class classification, for the short-time mode, relatively better accuracies were obtained, especially in the Word-Silent classification. This means that the pattern of brain waves, when it is busy with a word imagination (or perhaps any other), the task is much different from its background (idle) processes. So, if the subject cannot concentrate on his/her task, then the system performance will degrade in this (and generally all) type of classification. In the Word-Word classification, the large variance (20%) is due to the high correlation of EEG signals in too close time intervals, at least with some different but near tasks. This is a major shortcoming of the most imagined speech BCIs.

Finally, in the mixed-time mode, the best accuracies were obtained because in this mode we did not use both too far and too near signals.

Since the chance level was 50% in the 2-class, 33.3% in 3-class, and 14.3% in 7-class (balanced) classification, all accuracies were meaningfully above random classification (*p* value < 0.05), even in the worst case of the long-time mode.

In other modes, the accuracies, if not better, were comparable with the best reported values. For example, AlSaleh et al. reported an average maximum accuracy equal to 87.4% for 5 words classification over 9 subjects [32]. According to the reported data accusation schema, their classification method belonged to Mixed-time mode defined in this paper. Therefore, this value should be compared with 92 or 88 percent accuracies of 3 and 7-class types in Table 6.

DaSalla et al. had maximum accuracy about 78% for two class (vowel-silence) with three subjects [11]. In a similar manner, Brigham and Kumar achieved 68.8% for two syllables and seven subjects [16], compared to 74.5% for two words classification in our work.

Cooney et al. have published the results of their work on binary classification of word pairs [33]. Two different convolutional neural networks (CNN) and a baseline linear discriminant analysis (LDA) classifier were examined. 15 word pairs were constructed from six Spanish words of the EEG data set provided by Coretto et al. [34]. Despite of much smaller acquisition time, the data set can be considered as our long-time mode. Accuracies were 62.37% and 60.88% for deep and shallow CNNs and 57.80% for LDA classifier compared with 59.5% in our work. Also D-Y. Lee et al. reported 45% accuracy for the classification of the six words in Coretto's data set [22].

In another recent work using deep learning approaches, Panachakel et al. reported an average classification accuracy of 57.15% on 11 prompts of the KaraOne data set [35]. The prompts include seven phonemic/syllabic prompts (iy, uw, piy, tiy, diy, m, n) and four words (i.e., pat, pot, knew, and gnaw). Each prompt was presented 12 times for a total of 132 trials [36]. The recording time span was 30 to 40 minutes for each subject and therefore the classification is more similar to our short-time multiclass mode with 55.2% accuracy.

## 5. Conclusion

In this paper, we reported the results of a simple and extensible multi-class classifier working on self-recorded EEG signals during silent speech. Six complete and useful Persian words were the main classes, with the rest or silence for detecting talking/not-talking states. Although it seems that the language by itself may not change the way of human brain processing during speech, exploring Persian words in silent talk is new to literature in this domain of research. Also, the use of frequency spectrum as a feature and SVMs as the classifier have been reported in some papers, but the combination of this feature set (normalized to a constant length regardless of signal durations and sampling rates) and the purposed classifier has not been seen in previous studies.

Introducing classification modes, according to the time difference of EEG signals, is a new idea for discussing different accuracy values obtained in this and other related scholarly explorations. We made an attempt to expand this concept drawing on many other papers, which worked on imagined speech classification.

## Figures and Tables

**Table 1:** Class words and their class number.

Class word	bʌlʌ	bæle	chæp	kheɪr	pʌyɪn	rʌst	Silence
English equivalent	Up	Yes	Left	No	Down	Right	Silence
Abbreviation	U	Y	L	N	D	R	S
Class no (whole data set)	1	2	3	4	5	6	7
Class no (single records)	10…19	20…29	30…39	40…49	50…59	60…69	70…79

**Table 2:** Feature extraction parameters.

Parameter	Value
EEG channels	19 channels (all except A1 and A2)
Frequency range	1 to 32 Hz
T_w_	1 second
T_s_	2.5 seconds

**Table 3:** Word-Silence classification accuracies in all the three modes.

Classification mode	Classification accuracy (mean ± standard deviation)
Short-time	95.7 ± 8.3
Long-time	57.5 ± 7.2
Mixed-time	97.1 ± 5.1

**Table 4:** Results of classification in Word-Word case.

Classification mode	Classification accuracy (mean ± standard deviation)
Short-time	74.5 ± 20.4
Long-time	59.5 ± 7.5
Mixed-time	96.4 ± 8.0

**Table 5:** Results of classification in Word-Word-Silence case.

Classification mode	Classification accuracy (mean ± standard deviation)
Short-time	78.9 ± 13.9
Long-time	39.5 ± 5.4
Mixed-time	92.0 ± 11.1

**Table 6:** Summary of average accuracies in all classification types and modes.

Classification mode	Classification accuracy (mean ± standard deviation)			
	Word-Silence	Word-Word	Word-Word-Silence	6 Words+Silence
Short-time	95.7 ± 8.3	74.5 ± 20.4	78.9 ± 13.9	55.2 ± 8.8
Long-time	57.5 ± 7.2	59.5 ± 7.5	39.5 ± 5.4	32.0 ± 2.1
Mixed-time	97.1 ± 5.1	96.4 ± 8.0	92.0 ± 11.1	88.2 ± 4.8

## Data Availability

EEG data can be accessed by direct request from the corresponding author via mohammadkhani@irost.ir.
